# A review on the diagnosis and treatment of patients with clinically nonfunctioning pituitary adenoma by the Neuroendocrinology Department of the Brazilian Society of Endocrinology and Metabolism

**DOI:** 10.1590/2359-3997000000179

**Published:** 2016-08-01

**Authors:** Leonardo Vieira, Cesar L. Boguszewski, Luiz Antônio de Araújo, Marcello D. Bronstein, Paulo Augusto C. Miranda, Nina R. de C. Musolino, Luciana A. Naves, Lucio Vilar, Antônio Ribeiro-Oliveira, Mônica R. Gadelha

**Affiliations:** 1 Hospital Universitário Clementino Fraga Filho Universidade Federal do Rio de Janeiro Rio de Janeiro RJ Brasil Serviço de Endocrinologia, Hospital Universitário Clementino Fraga Filho, Universidade Federal do Rio de Janeiro (HUCFF-UFRJ), Rio de Janeiro, RJ, Brasil; 2 Hospital Federal da Lagoa Rio de Janeiro RJ Brasil Serviço de Endocrinologia, Hospital Federal da Lagoa, Rio de Janeiro, RJ, Brasil; 3 Hospital de Clínicas Universidade Federal do Paraná Curitiba PR Brasil Serviço de Endocrinologia e Metabologia, Hospital de Clínicas, Universidade Federal do Paraná (SEMPR), Curitiba, PR, Brasil; 4 Centro de Endocrinologia e Diabetes de Joinville Joinville SC Brasil Centro de Endocrinologia e Diabetes de Joinville (Endoville), Joinville, SC, Brasil; 5 Hospital das Clínicas Faculdade de Medicina Universidade de São Paulo São Paulo SP Brasil Serviço de Endocrinologia, Hospital das Clínicas, Faculdade de Medicina da Universidade de São Paulo (FMUSP), São Paulo, SP, Brasil; 6 Santa Casa de Belo Horizonte Belo Horizonte MG Brasil Serviço de Endocrinologia e Metabologia, Santa Casa de Belo Horizonte, Belo Horizonte, MG, Brasil; 7 Hospital das Clínicas Faculdade de Medicina Universidade de São Paulo São Paulo SP Brasil Divisão de Neurocirurgia Funcional, Instituto de Psiquiatria do Hospital das Clínicas, Faculdade de Medicina da Universidade de São Paulo (IPq-HC-FMUSP), São Paulo, SP, Brasil; 8 Hospital Universitário de Brasília Universidade de Brasília Brasília DF Brasil Serviço de Endocrinologia do Hospital Universitário de Brasília, Universidade de Brasília (UnB), Brasília, DF, Brasil; 9 Hospital das Clínicas Universidade Federal de Pernambuco Recife PE Brasil Serviço de Endocrinologia, Hospital das Clínicas, Universidade Federal de Pernambuco (UFPE), Recife, PE, Brasil; 10 Hospital das Clínicas Universidade Federal de Minas Gerais Belo Horizonte MG Brasil Serviço de Endocrinologia, Hospital das Clínicas, Universidade Federal de Minas Gerais (UFMG), Belo Horizonte, MG, Brasil; 11 Instituto Estadual do Cérebro Paulo Niemeyer Rio de Janeiro RJ Brasil Unidade de Neuroendocrinologia, Instituto Estadual do Cérebro Paulo Niemeyer, Rio de Janeiro, RJ, Brasil

**Keywords:** Clinically nonfunctioning pituitary adenoma, sellar mass, transsphenoidal surgery, radiotherapy, cabergoline

## Abstract

Clinically nonfunctioning pituitary adenomas (NFPA) are the most common pituitary tumors after prolactinomas. The absence of clinical symptoms of hormonal hypersecretion can contribute to the late diagnosis of the disease. Thus, the majority of patients seek medical attention for signs and symptoms resulting from mass effect, such as neuro-ophthalmologic symptoms and hypopituitarism. Other presentations include pituitary apoplexy or an incidental finding on imaging studies. Mass effect and hypopituitarism impose high morbidity and mortality. However, early diagnosis and effective treatment minimizes morbidity and mortality. In this publication, the goal of the Neuroendocrinology Department of the Brazilian Society of Endocrinology and Metabolism is to provide a review of the diagnosis and treatment of patients with NFPA, emphasizing that the treatment should be performed in reference centers. This review is based on data published in the literature and the authors’ experience. Arch Endocrinol Metab. 2016;60(4):374-90

## INTRODUCTION

Adenomas represent the most common primary neoplasm of the anterior pituitary, constituting 10-15% of all intracranial tumors (1). They are benign neoplasms of monoclonal origin, and their pathogenesis is thought to involve mostly inactivating mutations in tumor suppressor genes or activating mutations in proto-oncogenes, although the specific mutations remain to be identified in most cases (2).

Pituitary adenomas are divided into functioning and non-functioning according to the presence or absence of clinical syndromes resulting from hormonal hypersecretion. Upon imaging, these tumors can be further classified as microadenomas (< 10 mm) or macroadenomas (≥ 10 mm) according to size. Approximately 30% of pituitary adenomas are clinically nonfunctioning, and the majority of patients seeks medical attention for signs and symptoms resulting from mass effect on surrounding structures, i.e., the optic chiasm, cranial nerves and pituitary stalk. However, some cases may be diagnosed incidentally through imaging studies performed for other purposes (3). In this statement, the authors will provide a review on the diagnosis and treatment of patients with nonfunctioning pituitary adenomas (NFPA) whose presentation was not incidental.

The precise prevalence of NFPA is difficult to define because many patients are asymptomatic. The prevalence of clinically manifest NFPA is estimated at 7-22 cases per 100,000 inhabitants (4,5); its estimated incidence is 1 case per 100,000 inhabitants/year (6), with a peak incidence between the fourth and sixth decades of life with no sex predilection (7). Thus, it is estimated that there are approximately 2,000 new cases of NFPA diagnosed annually in Brazil (base for calculation: 200 million inhabitants).

The definition of NFPA is essentially clinical until histopathological analysis is performed, i.e., benign pituitary tumors that are not associated with clinical manifestations of hypersecretion of pituitary hormones (3). Most of these tumors are capable of producing glycoprotein hormones or their subunits in sufficient quantities to be detected by immunohistochemistry (IHC), *in situ* hybridization or molecular biology techniques; however, they cannot secrete detectable amounts of glycoprotein hormones in the circulation or secrete biologically inactive forms (8-14). Nevertheless, the α subunit of glycoprotein hormones may be elevated in the circulation in approximately 30% of NFPA patients (15). Although most of the gonadotroph adenomas are hormonally silent, rarely they secrete gonadotropin and may produce specific endocrine syndromes, such as ovarian stimulation-like syndrome in pre-menopausal women (16,17).

NFPA represents a heterogeneous group of adenomas and may be classified as gonadotropinomas, silent tumors that express only one pituitary hormone [adrenocorticotropin hormone (ACTH); thyroid-stimulating hormone (TSH), prolactin and growth hormone (GH)], multiple pituitary hormones (silent adenoma subtype 3) or no hormone (null cell) based on hormone expression on immunohistochemical examination. Most NFPA stems from gonadotropic cells, with immunostaining for follicle-stimulating hormone (FSH), luteinizing hormone (LH) and/or alpha subunit of glycoprotein hormones (14). Among silent corticotropinomas, silent adenoma subtype 1 (densely granulated) and subtype 2 (sparsely granulated) can be found (8,13,14). Table 1 shows the pathological classification of NFPA.

**Table 1:** Pathological classification of clinically nonfunctioning pituitary adenomas

Tumor subtype	Hormone expression	Frequency
Gonadotropinoma	FSH, LH, α subunit	68%
Silent adenomas		5%
Silent somatotropinoma	GH	
Silent prolactinoma	PRL	
Silent thyrotropinoma	TSH	
Silent corticotropinoma	ACTH	
Null cell	None	27%
Silent adenoma type 3	Multiple pituitary hormones	<1%

FSH: follicle-stimulating hormone; LH: luteinizing hormone; GH: growth hormone; PRL: prolactin; TSH: thyroid-stimulating hormone; ACTH: adrenocorticotropic hormone.

As to the importance of the characterization of NFPA in relation to the expression of pituitary hormones, some studies have found a relationship between the expression of hormones by IHC and tumor aggressiveness. In a study of 213 patients with NFPA, Yamada and cols. (18) found cavernous sinus invasion in 83% of silent corticotropinomas, 67% of adenomas subtype 3, 38% of null cell adenomas and 11% of gonadotropinomas, demonstrating differences in tumor invasiveness among NFPA subtypes.

In this publication, the goal of the Neuroendocrinology Department of the Brazilian Society of Endocrinology and Metabolism is to provide a review on the diagnosis and treatment of patients with NFPA, emphasizing that the treatment should be performed in reference centers. Thus, based on data published in the literature and the experience of the authors, representatives of the main reference centers for the treatment of pituitary adenomas in Brazil, we present a review for the diagnosis, treatment and follow-up of this disease.

## DIAGNOSIS

### Clinical presentation

The absence of clinical symptoms of hormonal hypersecretion contributes to the late diagnosis of the disease. Thus, the majority of patients seeks medical attention for signs and symptoms resulting from mass effect produced by a macroadenoma. NFPA can be manifested by symptoms of compression of the tumor on the normal gland and/or on the surrounding structures to the pituitary gland, such as the optic chiasm, cranial nerves, or pituitary stalk, causing hypopituitarism, hyperprolactinemia or, less frequently, pituitary apoplexy. Additionally, some cases may be diagnosed incidentally through imaging studies performed for other purposes, such as the evaluation of traumatic brain injury (12,19). The forms of clinical presentation of NFPA are found in Chart 1.

**Chart 1:** Forms of presentation of clinically nonfunctioning pituitary adenomas

• Mass effect (neuro-ophthalmologic complaints, neurologic, hypopituitarism and hyperprolactinemia by stalk effect)
• Pituitary apoplexy
• Incidental finding (pituitary incidentaloma)

NFPA with suprasellar expansion can reach and cause compression of the optic chiasm, determining varied visual impairment according to the involvement of nerve fibers. The most classic change is bitemporal hemianopia, but the visual loss can be uni-, bilateral or even central and is usually asymmetric and insidious. These visual changes can be detected by confrontation visual field testing or by visual field perimetry, preferably manual (Goldmann) (20). The suprasellar extension can produce headaches or, more rarely, intracranial hypertension, as large tumors may obstruct the foramen of Monro, leading to hydrocephalus (21). Headaches may be related to various factors, such as increased intra-sellar pressure, peritumoral inflammatory reaction, in addition to release of local neurotransmitters (22,23).

The invasion of the cavernous sinus (parasellar expansion) may affect the cranial nerves, causing a varied clinical profile according to the compromised nerve: eyeball shift out and/or ptosis (III nerve lesion, oculomotor nerve), deviation of the eyeball superiorly and slightly inward (IV nerve involvement, trochlear nerve) and convergent strabismus (lesion VI nerve, abducens nerve). More rarely, trigeminal neuralgia (lesion of branches V1 and/or V2 of V nerve, the trigeminal nerve) may occur (24).

More rarely, the tumor may be very aggressive and compress other intracranial structures, resulting in symptoms such as temporal lobe epilepsy or hydrocephalus. Cerebrospinal fluid (CSF) rhinorrhea can occur if the tumor causes erosion to the sellar floor and extends inferiorly (infrasellar extension) to the sphenoid sinus (12).

The deficiency of at least one pituitary hormone is generally present in most patients with macroadenoma and results from the compression of the normal anterior pituitary and/or pituitary stalk, preventing the stimulation of pituitary cells by hypothalamic factors and thus affecting the secretion of pituitary hormones. Hypogonadism, one of the most common deficiencies, can result from either a compressive effect on gonadotropic cells or the pituitary stalk or by non-tumoral hyperprolactinemia. In patients with NFPA, hyperprolactinemia occurs by compression of the pituitary stalk, which prevents the arrival of dopamine to the anterior pituitary, the main inhibitor of prolactin secretion, characterizing the hypothalamic-pituitary disconnection (stalk effect) (25-28).

Apoplexy is an acute vascular event that presents with acute expansion of tumor volume, manifesting itself by sudden onset of intense headache. It may be associated with neuro-ophthalmologic signs and symptoms, intracranial hypertension and altered levels of consciousness. In addition, it can cause hypopituitarism, including adrenal insufficiency, which can exacerbate the patient’s clinical status. The real risk of apoplexy in NFPA is not known because most of the cases reported the apoplexy was the clinical picture that led to the diagnosis of NFPA (29). Apoplexy is reported in 4-10% of cases in surgical series (25,27,28). As described in a review article (30), follow-up studies of patients with the incidental finding of nonfunctioning lesions likely to be NFPA reported an incidence of apoplexy in macroadenomas of 1.1% per year.

Currently, due to the increased availability of cranial computerized tomography (CT) and magnetic resonance imaging (MRI) in investigations of several neurologic disorders, there have been an increasing number of NFPA diagnoses in asymptomatic patients or those without signs suggesting pituitary pathology. In this situation, the NFPA is called an incidentaloma, which represents approximately 10% of clinical presentations among patients with NFPA (1,25,27,31,32).

Table 2summarizes the main clinical and laboratory characteristics of patients with NFPA evaluated in large studies (25-28). Growth hormone deficiency was not systematically assessed in most series; however, one study showed somatotrophic deficiency in 77% of cases (33).

**Table 2:** Clinical and laboratory characteristics of patients with clinically nonfunctioning pituitary adenoma

Clinical characteristics	Frequency
Headache	10 – 41%
Visual impairment	31 – 68%
Alteration of cranial nerve	4 – 5%
Incidental finding	7 – 12%
Hyperprolactinemia	28 – 54%
Apoplexy	4 – 10%
Hypopituitarism – documented	62 – 85%
Hypogonadism	43 – 78%
Hypocortisolism	24 – 32%
Hypothyroidism	20 – 25%

### Supplementary tests and differential diagnosis

NFPA diagnosis is based on the presence of sellar mass identified by CT, or preferably, MRI of the sella turcica, in the absence of signs and/or clinical symptoms of hormonal hypersecretion.

After the diagnosis of sellar mass, it is necessary to assess pituitary function with regard to hypersecretion or hypopituitarism. In the case of NFPA, the absence of hormone hypersecretion should be based on clinical evaluation and laboratory test analysis through the measurement of insulin-like growth factor type I (IGF-I) and prolactin (34,35). Tests for screening hypercortisolism include overnight 1-mg dexamethasone suppression test, Liddle 1 (suppression with low dose dexamethasone – 0.5 mg every 6 hours for 48 hours), late-evening salivary cortisol and urinary free cortisol. These tests are indicated only for patients in whom there is a clinical suspicion of Cushing’s disease (12,36).

The hook effect, an artifact of the dosage of the prolactin assay, occurs when excessively high levels of this hormone interfere with the formation of the complex antibody-antigen-antibody sandwich (immunometric method). The final effect is the detection of a signal lower than that expected by the test, erroneously determining a lower level of prolactin. To avoid this problem, prolactin assessment must be repeated at a serum dilution of 1:100. If the pituitary adenoma is a prolactinoma, prolactin levels will dramatically increase (proportional to tumor size). However, if the sellar lesion is not a prolactinoma, but an NFPA (or other sellar lesion), prolactin levels remain at a similar level after dilution (hyperprolactinemia due to hypothalamic-pituitary disconnection). Modestly elevated levels of prolactin (usually not exceeding 150-200 ng/mL) may occur in patients with macroadenomas or other sellar lesions by loss of inhibitory tone due to disruption of dopamine input on lactotrophs, configuring the stalk effect or hypothalamic – pituitary disconnection. Thus, in patients with macroadenoma, especially those > 3 cm and with normal or not so elevated prolactin values, a new dosage after a prolactin serum dilution of 1:100 must be obligatorily performed to distinguish prolactinoma from NFPA (34,37,38).

Cystic prolactinomas constitute another potential cause of discrepancy between the prolactin serum level and tumor volume since, although the tumor volume is large, the number of viable cells is low due to cystic degeneration.

NFPA, especially macroadenomas, can determine impairment of pituitary function by the involvement of gland or pituitary stalk compression; thus, hormone deficiencies should be investigated regardless of symptoms (35). The risk of hypopituitarism is directly related to tumor size; therefore, laboratory investigations should be performed in all patients with nonfunctioning macroadenomas. In patients with nonfunctioning microadenomas, the evaluation of pituitary function is suggested for lesions between 6-9 mm because these appear to be more closely associated with hypopituitarism (35). The assessment of pituitary function should be performed by the following hormonal dosages: 1) Corticotrophic axis through baseline cortisol levels (between 7:00am and 9:00 am) or stimulation test (synthetic ACTH or insulin tolerance test). Baseline cortisol levels < 3 mg/dL confirm adrenal insufficiency, while levels > 18 mg/dL exclude it. Cortisol values between 3-18 mg/dL require a stimulation test. Cortisol levels > 18 mg/dL during the stimulation test exclude the diagnosis of adrenal insufficiency [for details on the diagnosis of adrenal insufficiency see reference #(39)]; 2) Thyrotrophic axis through TSH and free T4 measurements. Although the TSH level is not informative in cases of central hypothyroidism (may be low, inappropriately normal or slightly elevated), a frankly high level indicates primary hypothyroidism, especially if accompanied by antithyroid autoantibodies; 3) Gonadotropin axis by measuring levels of LH, FSH and, in men, testosterone. In women, the determination of gonadotropin levels and menstrual cycles is sufficient for the evaluation of gonadal function. Inappropriately normal or low levels of gonadotropins in menopausal women indicate hypopituitarism (gonadotropin deficiency); and 4) Somatotrophic axis through IGF-I measurement. Importantly, normal IGF-I levels in adults with pituitary adenoma do not exclude the diagnosis of GH deficiency because nearly half of adult patients with GH deficiency may have normal levels of IGF-I (40). Nevertheless, in the presence of three pituitary hormone deficiencies, low IGF-I values are specific predictors of GH deficiency (41,42). If at least three deficiencies do not occur, GH liberation testing should be performed. Additionally, IGF-I levels may be low due to diseases, such as hepatic or renal impairment, uncontrolled diabetes mellitus, malnutrition and hypothyroidism (43).

Visual deficit, especially bitemporal hemianopia due to compression of the optic chiasm, can also occur (35). Thus, it is mandatory to perform visual field perimetry, preferably the Goldmann method, in all patients with macroadenoma with suprasellar expansion when the adenoma is very close (< 1 mm) or compresses the optic chiasm. Confrontational visual field performed by the endocrinologist in the outpatient clinic may not be precise whenever visual field perimetry is indicated. Thus, a formal visual field perimetry is needed.

In the analysis of sellar region imaging, preferably by MRI, it is important that an experienced neuroradiologist is on the team to set the adenoma size (microadenoma *vs.* macroadenoma); evaluate if there is extrasellar extension of the tumor, optic chiasm compression and/or sellar floor erosion (in this case, CT view is better to view erosion of the sellar floor); as well as aid in the differential diagnosis of masses that can affect the sellar region. MRI at diagnosis can already provide evidence of aggressive tumors, such as voluminous adenomas with an important extrasellar component, compression of adjacent structures and bone destruction. Pituitary adenomas usually present hypo- or isointense compared to normal pituitary tissue in T1 images on MRI. After administration of gadolinium, adenomas often present low contrast uptake, while the contrast enhancement in normal pituitary tissue occurs earlier (44).

Several other pathologies may be the cause of sellar mass and must be considered in the differential diagnosis (Table 3), although this distinction is difficult in many cases because the clinical presentations and radiological aspects may be similar (45,46). In this scenario, the role of the experienced neuroradiologist is crucial in the differential diagnosis of sellar masses. In other cases, some clinical, endocrinal and radiological findings of patients with a non-pituitary sellar or parasellar mass are present and help in this differentiation [for details on the other sellar lesions, see reference #(47)]. The correct etiologic diagnosis is important because the treatment of choice for many of these sellar and parasellar masses differs from that of pituitary adenomas.

**Table 3:** Differential diagnosis of major lesions that affect the sellar region

**Pituitary tumors**
• Prolactin-secreting pituitary adenoma
• Clinically nonfunctioning adenoma
• Growth hormone-secreting pituitary adenoma (acromegaly)
• Corticotropin-secreting pituitary adenoma (Cushing’s disease)
• Thyrotropin-secreting pituitary adenoma (TSHoma)
• Carcinoma*
**Other tumors**
• Craniopharyngioma
• Meningioma
• Germ cell tumor (germinoma, dysgerminoma and choriocarcinoma)
• Teratoma
• Gliomas
• Chordoma
• Metastasis (mainly: breast, lung and kidney)
**Cysts**
• Cyst of Rathke’s pouch
• Arachnoid cyst
• Others: epidermoid cyst and dermoid cyst
**Infiltrative/inflammatory lesions**
• Lymphocytic hypophysitis
• Sarcoidosis
• Langerhans cell histiocytosis
• Wegener´s Granulomatose
**Infectious diseases**
• Bacterial
• Tuberculosis
• Syphilis
• Deep mycosis
• Opportunist disease
**Vascular lesions**
• Aneurysms of the internal carotid artery
**Miscellaneous**
• Hypothalamic hamartoma
• Pituitary hypertrophy/hyperplasia

TSH: thyroid stimulating hormone. * Pituitary carcinoma is defined when there is the presence of distant metastases.

The presence of diabetes insipidus (DI) is common in tumors of non-pituitary origin and indicates that the sellar mass is most likely not a pituitary adenoma (45). Likewise, it is unlikely to diagnose a tumor that reduces light of the internal carotid artery by the cavernous sinus invasion as a pituitary adenoma (48). The dosage of the α subunit of the glycoprotein hormones can aid in the differential diagnosis, but it is elevated in only 30% of NFPA patients (15). Thus, high levels of α subunit indicate pituitary origin of mass, but normal values do not rule out NFPA as an etiologic diagnosis. Importantly, the α subunit is high in physiological situations such as menopause and pregnancy.

The definitive diagnosis of NFPA and the definition of its subtype are performed by histopathological analysis of the surgical specimen (12,49). Thus, patients not undergoing surgery remain with a presumptive diagnosis of NFPA. It is important to remember that approximately 90% of sellar masses are pituitary adenomas, and only 10% are lesions of non-pituitary origin, most of which are craniopharyngiomas and Rathke’s cleft cysts (50). Thus, a histopathological exam is needed to confirm pituitary adenoma; whenever possible, IHC should be performed for pituitary hormones, Ki-67, a marker of cell proliferation, and protein p53.

The summary for NFPA diagnoses is shown in Chart 2 and for requesting additional tests at diagnosis in Chart 3.

**Chart 2:** Summary for the diagnosis of clinically nonfunctioning pituitary adenoma

• Hormone hypersecretion evaluation should be based on laboratory test analysis through IGF-I and prolactin
• Special attention should be given to interpretation of plasma prolactin values because normal or slightly elevated values (hook effect) may occur in the macroprolactinomas, especially those > 3 cm, as well as prolactin levels until 150-200 ng/mL (hypothalamic-pituitary disconnection) can be seen in patients with NFPA. Thus, a new dosage after a prolactin serum dilution of 1:100 is mandatory to distinguish prolactinoma from a NFPA
• Hypercortisolism evaluation should be based on laboratory test analysis only in cases of clinical suspicion of Cushing’s syndrome
• Imaging exams of the sella turcica, preferably by MRI, to assess lesion size and its relationship with adjacent structures as well as assist in differential diagnosis with other sellar masses. In this context, the participation of an experienced neuroradiologist is essential
• In patents submitted to surgery, histopathological exam is needed to confirm pituitary adenoma

IGF-I: insulin-like growth factor type I; NFPA: nonfunctioning pituitary adenoma; MRI: magnetic resonance imaging.

**Chart 3:** Summary to request additional tests at diagnosis of clinically nonfunctioning pituitary adenoma

• Visual field perimetry, preferably Goldmann method, in patients with macroadenomas with suprasellar expansion that reach the optic chiasm
• The assessment of pituitary function must be performed in patients with lesions > 6 mm through dosages of cortisol, free T4, IGF-I, prolactin, LH, FSH, and in men, testosterone. In women, the determination of gonadotropin levels and menstrual cycles is sufficient for the evaluation of gonadal function

T4: thyroxin; IGF-I: insulin-like growth factor type I; FSH: follicle stimulating hormone; LH: luteinizing hormone; TSH: thyroid stimulating hormone.

## TREATMENT

In most cases, the treatment of NFPA patients requires a multidisciplinary approach involving the participation of endocrinologists, neurosurgeons, neuroradiologists, neuropathologists and radiation therapists. The established therapeutic approaches for patients with NFPA include observation (clinical follow-up), surgery and radiotherapy (RT).

In general, the main objectives of treatment of patients with macroadenomas are the preservation or restoration of visual and pituitary functions and long-term control of tumor growth.

### Surgery

The principal objective is decompression of the optical pathways and the preservation of adjacent structures as well as normal pituitary tissue. The success of surgical treatment varies directly with the surgeon’s experience and skill and inversely with the largest tumor diameter, its consistency, adhesion and degree of invasion (27,51-52). Experience is defined by the completion of at least 50 transsphenoidal surgeries annually (53). Thus, surgery should be performed in specialized reference centers.

Total resection of the tumor may be curative, which occurs most frequently in those with little or no extrasellar extension (7). In fact, the presence of cavernous sinus invasion and largest tumor diameter were negative predictors of complete tumor resection in some series (25,27,54). The total resection rate is highly variable, from 20-83% (mean approximately 50%), likely reflecting the advances in surgical and imaging techniques (particularly endoscopic technique) over time, as well as accumulated experience of neurosurgeons (25-28,33,55-58). Thus, about half of the patients are left with residual tumor after surgery.

Transsphenoidal surgery is the treatment of choice for patients with neuro-ophthalmologic symptoms because it is the only treatment modality capable of immediately determining decompression of the optical pathways. Visual improvement can be observed in up to 80% of patients (59). Thus, surgery should be indicated for patients with neuro-ophthalmologic complaints and/or tumors affecting the optic pathway. It is also indicated for patients with apoplexy who develop neuro-ophthalmologic complaints. In these cases, surgery is urgent (Chart 4). Patients with clinical presentation of apoplexy who are not operated on within a few days should undergo new imaging examination, even in cases with persistent visual loss due to tumor necrosis.

**Chart 4:** Summary for surgery

• Should be performed in reference centers for pituitary surgery
• Should be performed in patients with neuro-ophthalmologic complaints and/or tumors affecting the optical pathways, except in patients at high risk for surgery or who refuse the procedure
• Should be performed with urgency in patients with apoplexy who present neuro-ophthalmologic complaints

The recovery of pituitary function occurs in 30-50% of cases; however, recovery of adrenal and thyrotrophic function is more likely than somatotrophic or gonadotropic sectors (60). Losa and cols. (27) reported improvements in gonadal, thyroid and adrenal axes in 32.8%, 41.6% and 35.7%, respectively, and postsurgical worsening in 5.8%, 7.5% and 5.6% of patients, respectively. Nomikos and cols. (28) reported improvements in gonadal, thyroid and adrenal axes in 64.9%, 71.9% and 33.9% of patients, respectively, and postsurgical worsening in 2.1%, 4.5% and 1.5% of patients, respectively.

The transsphenoidal approach is the main surgical pathway, which has a low mortality rate when performed by skilled neurosurgeons (27,61-63). Major complications are rare and include worsening of visual field (0.5-2.4%), cerebrospinal fluid leak (1.5-4.2%), carotid artery lesion (0.4-1.4%), meningitis (0.5-1.9%) and hemorrhage (0.8-2.8%). Transitory diabetes insipidus can occur in up to 15% of surgeries, but its permanent form is less frequent (0.9-5%). The perioperative mortality rate is also low, ranging from 0.2 to 1.2% (61,62,64,65).

Adequate replacement of corticotrophic and thyrotrophic axes deficiencies before surgery is indicated, or at least, the glucocorticoid immediately before surgery in emergency cases to reduce surgical risk (66). The replacement should be kept in the postoperative period until further evaluation of all pituitary function is made.

Pituitary function and the visual field perimetry should be assessed 1-3 months after surgery and the treatment of hypopituitarism introduced according to hormone deficiencies. From the early postoperative period until the evaluation of pituitary function, special attention should be given to the corticotrophic axes for the risk of adrenal insufficiency. The assessment of pituitary function should be performed 1-3 months after surgery through dosages of cortisol, free T4, IGF-I, prolactin, LH, FSH, and in men, testosterone. In premenopausal women, determination of menstrual cycles is sufficient for the evaluation of gonadal function. The assessment of tumor resection extent should be made three months after surgery [three months is the minimum time for sellar MRI clearly showing a residual tumor, with less possibility of misinterpretation with postsurgical changes (surgical debris, for example)]. This MRI serves as the baseline imaging exam for comparison with the next MRI during follow-up (Chart 5).

**Chart 5:** Summary for outpatient postoperative evaluation

• Evaluation of pituitary function 1-3 months after the procedure - cortisol, free T4, IGF-I, prolactin, LH, FSH, and in men, testosterone. In premenopausal women, determination of menstrual cycles is sufficient to assess gonadal function
• Evaluation of the visual field through visual perimetry one month after surgery in patients who present initially with visual field defect
• Imaging exam of the sellar region, preferably MRI, at least 3 months after surgery

T4: thyroxin; IGF-I: insulin-like growth factor type I; FSH: follicle stimulating hormone; LH: luteinizing hormone; TSH: thyroid stimulating hormone; MRI: magnetic resonance imaging.

The long-term follow-up of patients undergoing surgery, but without receiving any type of adjuvant treatment, is detailed ahead.

### Radiotherapy

RT has been used as complementary treatment (adjuvant) to surgery to reduce the risk of tumor regrowth (67). Two modalities of RT are available, conventional and stereotactic, which can be fractionated or in single dose (radiosurgery).

Conventional RT is administered in fractionated doses four to five times a week for four weeks. There is no doubt of the effectiveness of RT in tumor control. Several studies have evaluated the impact of adjuvant conventional RT on tumor recurrence, and the progression-free rate is higher than 90% even in series with long-term follow-up (approximately 20 years) (68-71).

However, despite its effectiveness, there is no consensus on the systematic use of RT in the postoperative period in patients who remain with residual tumor. Nonetheless, the regrowth frequency is relatively high, occurring in approximately 40-50% of patients with residual tumor on imaging exams (26,33,54,56,71,72), with slow growth that is often insufficient to determine clinical manifestation (73). For some authors, if the remaining tumor is small and its evolution can be followed through periodic imaging exams (expectant approach), RT is only indicated when there is evidence of significant tumor growth (74). A recent meta-analysis showed that the tumor doubling time was 3.4 years, which means that in most adenomas, regrowth of residual tumor is a slow process and thus may not achieve sufficient dimensions to determine compressive symptoms (73). Furthermore, in 50-60% of cases, no growth is presented during follow-up (73).

To date, there are no markers to identify which tumors will present regrowth after surgery, making it difficult to decide which patients should undergo irradiation (75,76). Thus, indication for RT should be individualized, taking into account such issues as tumor characteristics on MRI at diagnosis, age, tumor doubling time, pituitary function, and fertility because the risk of hypopituitarism due to radiotherapy is high and grows over time with follow-up. In general, RT is reserved for cases of tumors not completely resected by surgery and that present progressive tumor growth during follow-up. Adjuvant RT should also be considered for patients who, at diagnosis, already present aggressive tumors (large adenomas with invasion and compromise to structures adjacent to sellar region) and/or tumors with pathologic characteristics that demonstrate aggressiveness, such as high mitotic index, Ki-67 > 3% and extensive immunostaining for p53 (atypical adenomas according to the classification of the 2004 World Health Organization) (49), and possibly silent corticotropinoma (77,78). Additionally, RT should be indicated in cases of persistent residual tumor after second surgery performed due to tumor recurrence. Finally, it can be used as primary therapy for patients with tumors proximate or reaching the optic pathway and concurrently present high surgical risk or surgery refusal (Chart 6). In this context, radiosurgery was effective in controlling tumor growth in 85% of patients in 10 years of follow-up (79). Patients undergoing RT should be monitored by MRI annually in the first five years and then every 2-3 years.

**Chart 6:** Summary for radiation therapy and monitoring

• Tumors that present progressive growth during follow-up
• Aggressive tumors on MRI at diagnosis and/or tumors with pathological characteristics that show aggressiveness
• Persistent residual tumor after second surgery for relapsing disease
• Primary therapy for patients with proximate tumors or that reach the optic pathways and high concomitant surgical risk or patient refusal for surgery
• Evaluation of hypopituitarism every 6 months
• Annual sellar MRI during the first five years and then every 2-3 years

MRI: magnetic resonance imaging.

Littley and cols. (80) evaluated 165 patients undergoing conventional RT; after five years of treatment, the incidence of GH deficiency, gonadotropin (LH and FSH), ACTH and TSH was 100%, 91% 77% and 42%, respectively. While observing different sensitivities between pituitary cells, the deficiency state can be unpredictable, which means the regular assessment of complete pituitary function in patients undergoing RT should be every 6 months. In addition, new deficiencies may appear up to 20 years after, justifying long-term follow-up of pituitary function (68). Evaluation for hypopituitarism should be performed every 6 months after RT in patients who have total or partial pituitary function preserved.

In addition to damage to the pituitary, there is a risk of optic neuropathy, especially to the optic chiasm (81), neurocognitive dysfunction (82), and more rarely, possible induction of other tumors of the central nervous system, especially meningiomas, astrocytomas and gliomas (83). It is important to consider that these complications occurred at times when the treatment was carried out with dated techniques, and risk has not yet been fully established with new RT techniques. In addition to the above side effects, studies have evaluated the relationship between RT with increased mortality, especially for cerebrovascular disease (84,85). Brada and cols. (85) found a relationship between radiation dose and the risk of cerebral infarction in a study with 331 patients. The relative risk of occurrence of a first stroke in relation to the general population was 4.1 in this study. Furthermore, hypopituitarism, as well as hormone replacement, is also related to cerebrovascular disease and increased mortality (86,87). Thus, increased mortality from cerebrovascular disease appears to be multifactorial, involving the direct effect of radiation on cerebral vessels, hypopituitarism and its treatment (88). Because risk of cerebrovascular disease is a long-term effect, the radiation used in the reported cases was from an older technique, where parts of the brain received up to half or a third of the total dose.

In an attempt to minimize the risks of RT, a stereotactic method was developed. This technique has successfully replaced conventional RT in controlling the growth of tumor remnants (89). In the study of Mingione and cols. (90), 59 of 90 patients (65.6%) with NFPA treated after surgery showed tumor volume reduction, the tumor remained stable in 24 (26.7%) and there was tumor growth in 7 cases (7.8%). Like conventional RT, the rate of tumor control of NFPA with stereotactic RT published in different series is above 90%, but with less follow-up (91). Mokry and cols. (92) reported an incidence of hypopituitarism of 20%, but it may have been underestimated by the follow-up time (28.9 ± 21.5 months) (93). In the series of Hoybye and cols. (94), hypopituitarism developed in 68% of patients with corticotropinoma at a mean follow-up of 17 years (12-22 years). As there is lesser existing experience with stereotactic irradiation in single or fractionated doses, more long-term studies are needed to assess the actual incidence of side effects, such as hypopituitarism, optic neuropathy, neurocognitive dysfunction, cerebrovascular disease, possible induction of other tumors of the central nervous system, and mortality (93).

### Medications

NFPA are tumors without an established drug treatment. Three classes of drugs have been studied for the treatment of NFPA: dopamine agonists (DA), somatostatin analogs (SA) and gonadotropin-releasing hormone (GnRH) analogues (95). Temozolomide has also been used in some centers for patients with aggressive tumors. However, to date, none of these are routinely used or formally indicated for the treatment of these tumors.

#### A – Dopamine agonists

DA was the first class of drugs used in the treatment of prolactinomas and somatotropinomas; bromocriptine (BRC) and cabergoline (CAB) are both commercially available DA in Brazil. These drugs act directly on the dopamine receptor type 2 (DR2), present in normal pituitary (96) and pituitary adenoma (96,97). The use of this class of drugs in the treatment of NFPA is based on the observation that these tumors express DR2 (96,98,99). Promising results have been reported on the efficacy of CAB in the control of tumor growth (98,99).

Pivonello and cols. (98) sought to correlate DR2 expression with the effect of CAB on tumor volume in patients with NFPA. The study included 18 patients undergoing surgical treatment, of which 9 (50%) remained with residual tumor and were treated with CAB for a period of 1 year. Significant tumor reduction (over 25%) was observed in 56% of patients. Among them, remarkable tumor reduction (greater than 50% of the initial volume) was identified in two patients, and moderate tumor reduction (25-50% of the initial volume) was observed in three patients. All patients with significant reduction of adenoma expressed DR2. A similar result was found by Vieira Neto and cols. (99), who observed tumor reduction of > 25% in 67% (6/9 patients) after 6 months of treatment with CAB. In both studies, the cabergoline dose was titered up to 3 mg/week or to the highest tolerated dose.

Greenman and cols. (100) evaluated the use of DA immediately after surgery or after detection of remnant tumor growth during follow-up. Thirty-three patients received DA during a mean period of 40 months and were matched with a group of 47 patients who were not treated with the drug. Tumor reduction or stability was observed in 18 of 20 patients (90%), when DA was started immediately after detection of the residual tumor on postoperative MRI. In 13 patients, treatment was started only after detection of remnant tumor growth during routine visits. In this group, reduction or stabilization of the tumor was detected in 8 of 13 patients (61.5%). Conversely, tumor size remained stable in only 18 of 47 patients (38.3%) not treated with DA, and tumor growth was detected in 29 patients (61.7%). Thus, based on these results, the authors concluded that DA treatment induces a reduction or stabilization of tumor volume, particularly when the treatment was initiated prior to detection of tumor relapse/regrowth.

Although CAB has not been included in the treatment algorithm of patients with NFPA, a therapeutic attempt can be made according to the clinical judgment in individual cases with postoperative residual tumor, especially those with extrasellar extension (regrowth predictor). In these patients, small growths may be clinically relevant in terms of compressive effects. In this context, CAB could be attempted before RT indication for control of tumor regrowth, avoiding the side effects related to radiation, especially in patients with preserved pituitary function.

The initial dose can be 0.5 mg weekly, increasing 0.5 mg each week until a maximum dose of 3.0 mg/week. If the patient has intolerance during titration, use the highest tolerated dose. The effectiveness of CAB should be assessed by MRI, which can be performed after 6 months of medication at a maximum/tolerated dose. It is not defined in the literature what is considered a response to CAB, given the heterogeneity of the studies evaluating its effect on tumor volume. Tumor stability may be considered a positive response; however, as mentioned above, 50-60% of patients will keep tumor stability even without treatment. Therefore, in cases with stability, it is not possible to infer the actual effect of the treatment, and individual analysis of cost/benefit should be made to decide the maintenance of medication in these cases. Similarly, there is no data on the withdrawal of CAB among patients who present a benefit from its use. According to the clinical judgment of the physician, if the patient has a benefit and no side effects, the drug can be maintained indefinitely. While using CAB, after the initial 6-month assessment of the use of the maximum/tolerated dose, MRI should be made annually. Because the impact of CAB on tumor volume is uncertain, and pituitary function may deteriorate due to tumor growth, pituitary function should be assessed regularly every 6-12 months for safety reasons. If the tumor presents progressive growth during the use of CAB, RT should be indicated. If the patient develops visual impairment or the tumor begins to compress visual pathways, further surgery is indicated.

In most studies, BRC has failed to induce tumor shrinkage (101-103). The compilation of data from seven studies on the effectiveness of BRC in the treatment of 84 patients with NFPA has shown no reduction in tumor volume in 76 patients (90%) and tumor increase in one case (1.2%) (101). In seven patients (8%), a small tumor reduction was observed, although one case evolved coincident with pituitary apoplexy. Visual deterioration was observed in five patients (6.6%), whereas only one patient showed improvement of the visual field (101).

Adverse reactions to CAB occurred in 13% of patients in a large series in which this drug was given (104) and included nausea, dizziness, headache, constipation, dry mouth, nasal obstruction and postural hypotension (105). Schade and cols. (106) showed a high risk of heart valve lesion in patients with Parkinson’s disease using CAB in doses much higher than those used for pituitary adenomas. These authors showed that this risk was dose- and time-dependent. Although clinically relevant valve lesion has not been reported in patients taking DA to treat prolactinomas (107), a transthoracic echocardiography should be performed as good clinical practice before CAB and then every 12 months during therapy in patients taking CAB at a dose > 2.0 mg/week (34,108). The drug should be discontinued if there is evidence of onset or progression of valve lesion.

#### B – Somatostatin analogues

SA exerts its pharmacological properties through SSTR, with five subtypes having been identified in humans (SSTR1, 2, 3, 4 and 5) and present in normal pituitary and pituitary adenoma (96,109-111).

The synthetic SA currently available in clinical practice, octreotide (OCT) and lanreotide (LAN), exert their action primarily by binding to SSTR subtypes 2 and 5 (112). The SSTR detection in NFPA raised the possibility that SA might be used in the treatment of this tumor type (109,113,114). However, the favorable effects of therapy with commercially available SA have been variable and infrequent, beyond correlating poorly with SSTR expression (115). Vieira Neto and cols. (114) observed that the most expressed receptor among NFPA was SSTR3, while SSTR2 expression was found to be low, which could explain the low efficacy of SA. Thus, we do not recommend these SA for the treatment of patients with NFPA.

**Figure 1:**
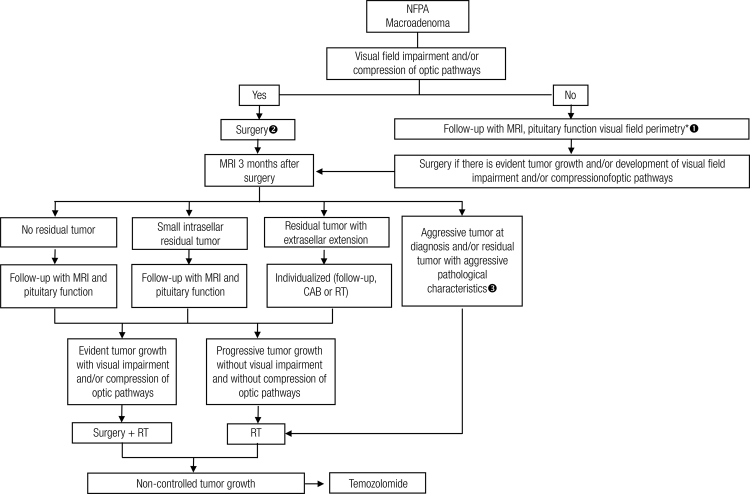
Management of patients with clinically nonfunctioning pituitary adenoma

NFPA: nonfunctioning pituitary adenoma; MRI: magnetic resonance imaging; CAB: cabergoline; RT: radiotherapy.

New SA with potential therapeutic impact on the management of patients with pituitary adenomas are under study. Pasireotide is a universal SA with action on SSTR subtypes 1, 2, 3 and 5. As pasireotide has action on SSTR3, the most expressed receptor in NFPA, it is possible that this drug could be an effective option in the therapeutic approach for NFPA patients. Studies evaluating its *in vivo* effect on tumor volume in patients with NFPA are under evaluation (Figure 1).

* For cases that can be followed up with MRI, are asymptomatic from a visual point of view and the tumor is located far from the optical pathways, there is no need for visual field perimetry.

^1^ For asymptomatic patients with no compression of the optic pathway but with tumors near the optic chiasm, the decision between surgery and follow-up should be individualized, taking into consideration issues such as age, fertility/pregnancy, pituitary function and the patient’s desire.

^2^ RT can be used as primary therapy for patients with tumors near or that reach the optic pathways and present surgical risk high enough to contraindicate surgery.

^3^ Tumors with pathologic features of aggressiveness are those with a high mitotic index, Ki-67 > 3% and extensive immunostaining for p53.

#### C – GnRH analogues

This class of drugs acts through receptors for GnRH, exerting their effects by saturation of these receptors in the gonadotropic cells, thereby reducing hormone production. Because some NFPAs are positive for gonadotropins, GnRH agonists and antagonists have been tested in the treatment of these adenomas. However, the use of GnRH agonist analogues had a null effect or exacerbated gonadotropin secretion with no change in tumor dimensions (10,116-118).

Thus, GnRH analogues are not recommended as medical therapy for NFPA because it does not provide tumor reduction and can eventually exacerbate hormone hypersecretion or even cause pituitary apoplexy, especially in large volume tumors (119).

#### D – Temozolomide

Temozolomide is an alkylating agent routinely used in the treatment of brain gliomas and metastatic melanomas that has a cytotoxic effect. It also acts by inhibiting angiogenesis in tumor tissues through an unknown mechanism. O6-methylguanine DNA methyltransferase (MGMT) is an enzyme that repairs DNA by removing the alkyl group and inducing resistance to temozolomide (120,121). Widhalm and cols. (121) showed that, in aggressive NFPA, low MGMT immunoreactivity was found in 50% of patients compared with 24% of patients with non-aggressive NFPA, suggesting that temozolomide may be a therapeutic option in those patients. The main side effect of the drug is the induction of lymphopenia (120). Treatment with temozolomide can be attempted in patients with aggressive tumors that did not achieve control of tumor growth despite RT and other therapeutic measures and in rare cases of patients with pituitary carcinoma.

## LONG-TERM FOLLOW-UP OF PATIENTS UNDERGOING SURGERY

### Postoperative follow-up of patients with no residual tumor

For patients with postoperative MRI with no evidence of residual tumor, the course of treatment must be clinical follow-up, with no need for adjuvant treatment, such as RT because recurrence occurs in approximately 10% of cases (26,33,54,56,72) (Chart 7). In fact, the meta-analysis performed by Chen and cols. (73) to evaluate recurrence after surgery found a recurrence rate of 12% (371 patients), with a progression-free survival rate of 96% and 82% at 5 and 10 years, respectively.

**Chart 7:** Postoperative follow-up of patients with no residual tumor

• The best approach is clinical follow-up
• Annual MRI in the first 5 years. In the absence of relapse during this period, MRI every 2-3 years
• Visual field perimetry should be performed only in patients who present tumor growth about to reach the optical pathway and/or determine visual impairment
• Evaluation of pituitary function should be performed every 6-12 months if MRI does not demonstrate relapse. If there is recurrence, evaluation of pituitary function should be performed at the time of detection

MRI: magnetic resonance imaging; RT: radiotherapy.

Follow-up for these patients should be performed with annual MRI in the first 5 years. In the absence of relapse during this period, MRI should be performed every 2-3 years. If recurrence is detected, management should be individualized. If growth is not clinically relevant and the patient remains asymptomatic, a new MRI should be performed in 6 months to detect if the tumor is in progressive growth. If this is the case and the patient does not have compressive complaints, RT is indicated. If the patient develops visual impairment or the tumor begins to compress visual pathways, further surgery is indicated.

Laboratory evaluation for hypopituitarism during follow-up should be performed every 6-12 months if MRI does not show recurrence. Laboratory evaluation for hypopituitarism should be performed during follow-up every 6-12 months because new deficiencies may reflect tumor growth/recurrence. To avoid much expenditure for the patient and the hospital, annual MRI scans might be combined with semi-annual laboratory evaluation because the intermediate blood tests could provide a way to detect new deficiencies before the next scheduled MRI scan. If there is recurrence, evaluation of pituitary function should be performed at the time of detection.

Visual field perimetry should be performed only in patients who present tumor growth about to reach the optical pathway and/or determine visual impairment. For patients who can be followed with MRI and are asymptomatic from a visual point of view, there is no need for regular visual perimetry.

### Postoperative follow-up of patients with residual tumor

As mentioned above, approximately half of patients with NFPA have residual tumor after surgery (25-28,33,55-58). For these patients, there is no consensus on the best approach. RT is highly effective in preventing tumor regrowth, but it is associated with significant long-term complications. Conversely, postponing any type of treatment is associated with progression (tumor regrowth) in approximately 40-50% of cases (26,33,54,56,71,72,122). Chen and cols. (73) showed that there was tumor regrowth in 46% of 600 patients with tumor remnants, with a progression-free survival rate of 56% and 40% at 5 and 10 years, respectively.

To date, there is no reliable marker to predict tumor regrowth after surgery. Pathological characteristics have been studied with the aim of analyzing the biological behavior of NFPA. In this scenario, IHC for Ki-67, a marker of cell proliferation, and p53 protein remains the most widely used (75). Although some studies have found a higher rate of Ki-67 labeling in invasive tumors (123-125), this result has not been replicated by others (18,126). Studies that evaluated the predictive value of the Ki-67 index for recurrence or regrowth of residual tumor were also inconsistent (76,127-131). The lack of unanimity between studies can be explained in part by the fact that the Ki-67 quantifies only the replication potential of the tumor and does not assess tumor growth, which results from the balance between proliferation and cell death (132). In general, high Ki-67 (> 3%) suggests a more aggressive tumor, whereas tumors with low Ki-67 (< 3%) may or may not exhibit aggressive behavior. Similarly, extensive immunostaining for p53 suggests a more aggressive tumor, whereas tumors that are IHC-negative for p53 may or may not exhibit aggressive behavior (125). Finally, pluri-hormone adenomas also appear to present a higher chance of recurrence or relapse (133), and silent corticotroph adenomas may also have more aggressive behavior (77,78).

Several large series evaluating postoperative recurrence/tumor regrowth have shown that the presence of residual tumor and/or follow-up duration appear to be the two major determinants of recurrence/regrowth (26,33,54,56,71,72). Dekkers and cols. (33) found that, among patients without residual tumor, the recurrence-free rate at 5 and 10 years was 100%. In patients with residual tumor, after a mean follow-up of 6.3 years (3-11 years), the progression-free rate at 5 and 10 years was 92% and 74%, respectively. In this study, follow-up time was a tumor regrowth predictor (33). In the series by O’Sullivan and cols. (56) the relapse rate among patients without residual tumor was 0% and 33.5% for those with residual tumor during median follow-up of 4.1 years (1-20.7 years), so the rate of progression at 5 and 10 years was 24.4% and 51.5%, respectively. In this study, follow-up duration and the presence of residual tumor with extrasellar extension [odds ratio (OR) 3.73] were predictors of regrowth (56). Greenman and cols. (54) similarly found that the presence of residual tumor and supra- and infrasellar extension were associated with an increased risk of regrowth. Thus, to date, it seems reasonable to use the presence of residual tumor after a successful surgery to select high-risk patients for possible medical intervention, leaving the low-risk patients (without residual tumor) under close surveillance (see above).

For patients with small residues (intrasellar) far from the optic chiasm, clinical follow-up (“wait and see policy”) seems to be the best approach since growth, when it occurs, is slow and insufficient to determine compressive symptoms (73). Follow-up should be performed with an annual MRI in the first 5 years. In the absence of regrowth during this period, MRI should be performed every 2-3 years. Importantly, comparisons should be made between the current image exam and the previous exam as well as all previous exams, as small growths cannot be detected by comparing only the last two imaging exams. If regrowth is detected, the approach should be individualized. If growth is not clinically relevant and the patient remains asymptomatic, with the tumor still far from the optical pathways, a new MRI should be performed in 6 months to detect if the tumor is in progressive growth. If the tumor growth is in progression but the patient is asymptomatic, RT should be considered. In this context, CAB may also be an option provided the risks and benefits are discussed with the patient. Greenman and cols. (100) offered DA therapy in a subset of patients with NFPA (n = 13) upon tumor expansion. DA therapy ceased tumor growth in six patients (46.1%) and determined tumor reduction in two patients (15.4%). Tumor growth persisted in five patients (38.5%) despite therapy. Thus, the overall control rate was 61.5% (8/13). On the other hand, in untreated control patients, tumor size remained stable in only 38.3% (18/47), whereas tumor growth was verified in 29 controls (61.7%). Moreover, in the control group there was no case of spontaneous tumor reduction. Therefore, the authors concluded that DA therapy induces a reduction or stabilization of tumor volume in patients with tumor relapse/regrowth. Based on these results, CAB could be attempted before RT indication for control of tumor regrowth, avoiding the side effects related to radiation, especially in patients with preserved pituitary function.

If treatment with CAB is introduced, MRI should be performed at 6 months to indicate whether there was stabilization/reduction of the lesion (which would justify the maintenance of medication) or tumor progression (which would indicate RT). If the patient develops visual impairment or the tumor begins to compress visual pathways, further surgery is indicated (Chart 8).

**Chart 8:** Postoperative follow-up of patients with residual tumor

• Patients with small residues (intrasellar) far from the optic chiasm should be submitted to clinical follow-up (“wait and see policy”)
• The follow-up should be with annual MRI in the first 5 years. In the absence of regrowth during this period, MRI every 2-3 years
• If regrowth is detected, the approach should be individualized
• If growth is not clinically relevant and the patient remains asymptomatic with the tumor still far from the optical pathways, a new MRI should be performed in 6 months to detect whether the tumor is in progressive growth
• If the tumor growth is in progression but the patient is asymptomatic, RT should be considered. CAB can also be an option
• If the patient develops visual impairment or the tumor begins to compress visual pathways, further surgery is indicated
• Evaluation of pituitary function every 6-12 months. For patients submitted to RT, evaluation of pituitary function should be performed every 6 months
• Annual visual field perimetry should be performed in patients with tumors with suprasellar extension or those whose tumor growth reaches the optical pathways and/or determines visual impairment

MRI: magnetic resonance imaging; RT: radiotherapy; CAB: cabergoline.

Laboratory evaluation for hypopituitarism during follow-up should be performed every 6-12 months. For patients submitted to RT, evaluation of pituitary function should be performed every 6 months.

Annual visual field perimetry should be performed in patients with tumors with suprasellar extension or those whose tumor growth reaches the optical pathways and/or determine visual impairment.
